# Clinical characteristics of 1124 children with epiphyseal fractures

**DOI:** 10.1186/s12891-023-06728-9

**Published:** 2023-07-21

**Authors:** Hansheng Deng, Zhenhui Zhao, Zhu Xiong, Futang Gao, Shengping Tang, Yuanheng Li, Weiqing Li, Jihuang Huang, Shuting Cui, Xiaodi Chen, Shuaidan Zeng, Gen Tang, Leonardo Antonio Sechi, Gianfilippo Caggiari, Carlo Doria, Xin Qiu

**Affiliations:** 1 Department of Biomedical Sciences, University of Sassari, 07100 Sassari, Italy; 2 Orthopaedic Department, Sassari University Hospital, 07100 Sassari, Italy; 3 Shenzhen Children’s Hospital of Shantou Medical University, Shenzhen, People’s Republic of China; 4 Shenzhen Children’s Hospital of China Medical University, Shenzhen, People’s Republic of China; 5 Department of Pediatric Surgery, Shenzhen Baoan Women’s and Children’s Hospital, Jinan University, Shenzhen, People’s Republic of China; 6 Shenzhen Institute of Artificial Intelligence and Robotics for Society, CAS Key Laboratory of Human-Machine Intelligence-Synergy Systems and the SIAT Branch, Guangdong Province, Shenzhen, People’s Republic of China; 7 Institutes of Advanced Technology (SIAT), Chinese Academy of Sciences, The Guangdong-HongKong-Macau Joint Laboratory of Human-Machine Intelligence-Synergy Systems, Shenzhen, Guangdong Province, Shenzhen, People’s Republic of China; 8 Department of Pediatric Surgery, Affiliated Hospital of Guangdong Medical University, Zhanjiang, People’s Republic of China

**Keywords:** Pediatric, Epiphyseal fracture, Epidemiology

## Abstract

**Background:**

In this study, to provide a theoretical basis for understanding the clinical characteristics of epiphyseal fractures in children and improving their management, we explored and analyzed the proportions of different types of epiphyseal fractures in children and evaluated the causes of injury and epidemiological characteristics.

**Methods:**

We retrospectively analyzed children younger than 18 years with fresh epiphyseal fractures who were admitted to our hospital from July 2015 to February 2020. Demographic information, injury mechanisms, fracture characteristics, fracture classification and surgical information were collected.

**Results:**

A total of 1124 pediatric patients (1147 epiphyseal fractures), including 789 boys and 335 girls, were included in this study. Epiphyseal fractures were classified as Salter-Harris type II (1002 cases), type IV (105 cases), type III (25 cases), Salter-Harris type I (14 cases), and Salter-Harris type V (1 case). The number of fracture sites peaked in the adolescent group (440 cases). The most three common sites of epiphyseal fractures were the distal radius (460 cases) in which Salter-Harris type II fractures were the most common (454 cases) and Salter-Harris type I (3 cases), Salter-Harris type IV (2 cases), Salter-Harris type III was the least common (1 case). Followed by phalanges of fingers (233 cases) in which Salter-Harris type II fractures were the most common (224 cases) and Salter-Harris type IV (4 cases), Salter-Harris type I (3 cases), Salter-Harris type III fractures were the least common (2 cases). Distal humerus (146 cases) in which Salter-Harris type II fractures were the most common (95 cases), followed by Salter-Harris type IV (49 cases), Salter-Harris type I fractures were the least common (2 cases). The most three important causes of fractures were falls (720 patients), car accident injuries (68 patients), and basketball falls (43 patients). Among the 1124 children with epiphyseal fractures, 1058 were treated mainly by surgery and the ratio of open and closed reduction was 1:5.3. Eighty-eight patients showed an interval > 72 h between the injury and the hospital visit. Among these 88 patients, the most common fracture type was distal radial epiphyseal fracture (32 cases), and all fractures were of Salter-Harris type II.

**Conclusions:**

The epidemiological characteristics of epiphyseal fractures in children indicate the need to strengthen health and safety education and protective measures to prevent the occurrence of these fractures in children. In addition, emergency surgeons and orthopedic surgeons in general hospitals should strengthen their basic knowledge of diagnosing and treating epiphyseal injuries in children to reduce missed diagnoses, misdiagnoses or malpractice.

## Introduction

The rapid advancements in science and technology and the popularization of various modern means of transportation have greatly improved living standards. However, these improvements have also increased the incidence of trauma caused by various energies. The incidence of fractures in children, who tend to show poor awareness of self-protection, has been consistently increasing, so that the epiphyseal and epiphyseal plate fractures are common bone injuries in childhood. Epiphyseal injuries accounted for 17.9% of all pediatric fractures in Japan [1–3]. The Salter-Harris classification is the most commonly used system for categorizing epiphyseal fractures in children [4]. The epiphyseal plate in children is a layer of weak cartilage tissue responsible for the longitudinal and lateral growth of long bones [5, 6]. It lacks blood supply and has poor regenerative ability. Therefore, improper treatment of epiphyseal fractures can have serious consequences, including bone bridge formation and premature epiphyseal plate closure, potentially causing shortened limbs and/or angular deformities and resulting in lifelong disabilities [7].

Epidemiological studies on epiphyseal fractures in Chinese children and adolescents are lacking. Therefore, we aimed to obtain the epidemiological data of young patients with epiphyseal fractures admitted to our hospital and provide a theory for reducing the incidence of epiphyseal fractures in children and improving the management of these fractures. These findings can be expected to provide a scientific basis for improving the quality of children’s life with epiphyseal fractures, formulating health education policies, and reducing the economic burden associated with these fractures.

## Methods

We retrospectively analyzed the findings for children with epiphyseal fractures who were hospitalized at Shenzhen Children's Hospital between July 2015 and February 2020 and extracted information regarding age, sex, injury cause, fracture site and type as well as data on methods of treatment, hydrology, the interval between injury and hospital visit, costs during hospitalization; the data were obtained within 14 days of the injury. The inpatients were grouped as follows by age: age ≤ 730 days, infants; 730 days < age ≤ 2190 days, preschool children; 2190 days < age ≤ 4015 days, schoolchildren; and 4015 days < age ≤ 6570 days, adolescents.

On the basis of the injury mechanism, we classified the first and second-level classification of the fracture etiologies (Table 1) [8]. The interval between injury and hospitalization was categorized as < 4 h, 4–6 h, 7–11 h, 12–23 h, 24–47 h, 48–72 h, 73 h–5 d, and 6–14 d, respectively.

**Table 1:** First and second-level classification of the fracture etiologies

First-level classification of the etiologies	Second-level classification of the etiologies
***Daily-life injuries***	
	falls
	crush injuries
	clipping
	furniture-related falls
	sprains
	bunk bed falls
	falls from height
	cuts
	strains
	twist injuries
***Road traffic injuries***	
	car accident injuries
	bicycle falls
	bicycle-spoke injuries
	falls from vehicles
***Sports injuries***	
	single/parallel bar falls
	basketball falls
	falls while running
	skateboard falls
	soccer falls
	kick injuries
	falls during physical education activities
	trampoline falls
	balance bike falls
	dance falls
	slide falls
	ice skating falls
	playground falls
	taekwondo falls
	jump rope falls
***Abuse injuries***	
***Unknown***	

## Results

### Age and sex

A total of 1124 children were included, including 789 boys and 335 girls. In all age groups, the number of boys with epiphyseal fractures was higher than the number of girls. The number of epiphyseal fractures was the lowest in the infant group and the highest in the adolescent group. The infant group had 81 patients with epiphyseal fractures, including 48 boys and 33 girls. With the growth and development of children, the number of epiphyseal fractures gradually increased and the number was the largest in the adolescent group (428 patients, including 360 boys). The adolescent group also included 68 girls with epiphyseal fractures, and it showed the highest male-to-female ratio (5.3:1). The most common cause of damage was falls (494 cases in boys and 226 cases in girls). Among the total amount of 1124 children, the average length of hospitalization was 4.71 ± 5.18 days, and the average hospitalization cost was 5557.68 RMB (interquartile range, 4514.55–7558.22 RMB; Table 2).

**Table 2:** Demographics of patients with 1124 fractures

Parameter	Patients n(%)
Numbers	1124
Age class	
Infants	81(7.21%)
Preschool children	238(21.17%)
School children	377(33.54%)
Adolescents	428(38.08%)
Sex	
Girl	335(29.80%)
Boy	789(70.20%)
Hospitalization expenses (RMB)	5557.68(IQR,4514.55–7558.22)yuan
Hospital stays (day)	4.71 ± 5.18

### Fracture site and classification

**Fig. 1:**
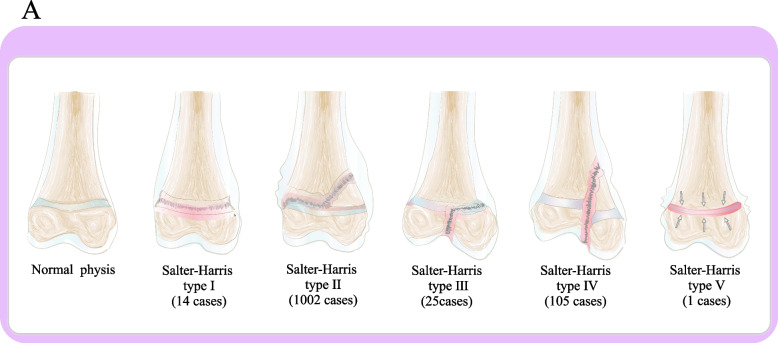
This picture shows the number of S–H fractures for all epiphyseal fractures

**Fig. 2:**
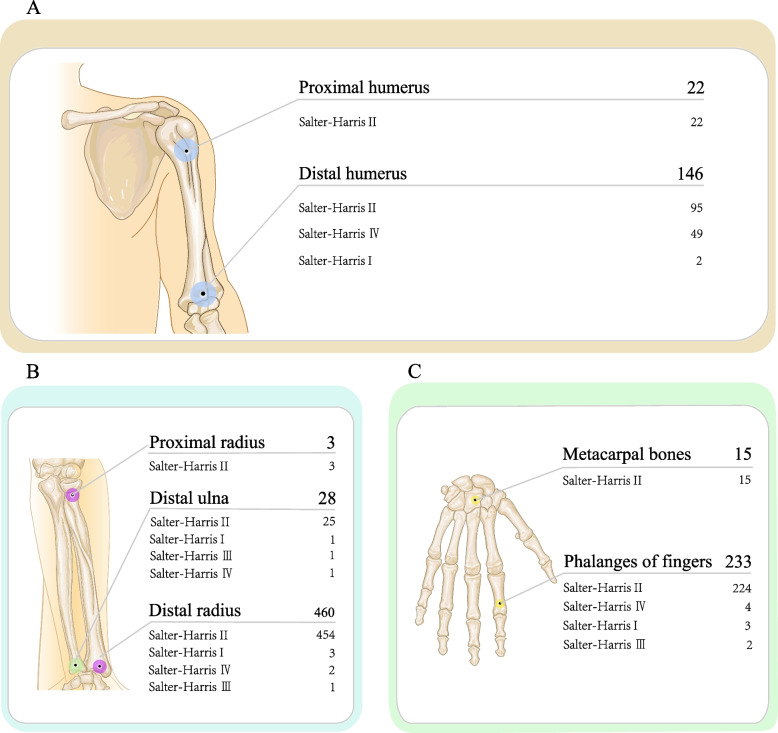
This picture shows the number of S–H fractures for Upper Extremity epiphyseal fractures

**Fig. 3:**
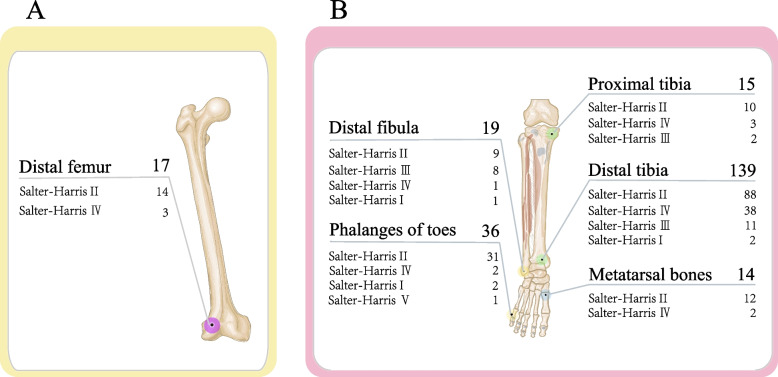
This picture shows the number of S–H fractures for Lower Extremities epiphyseal fractures

A total of 1147 epiphyseal fracture sites were recorded in the 1124 pediatric patients (Table 3). Among the 1147 epiphyseal fractures, Salter-Harris type II was the most common fracture type (1002 cases), including Distal radius (454 cases), Phalanges of fingers (224 cases), Distal humerus (95 cases), Distal tibia (88 cases), Phalanges of toes (31 cases), Distal ulna (25 cases), Proximal humerus (22 cases), Metacarpal bones (15 cases), Distal femur (14 cases), Metatarsal bones (12 cases), Proximal tibia (10 cases), Distal fibula (9 cases), Proximal radius (3 cases). Followed by Salter-Harris type IV (105 cases), including Distal humerus (49 cases), Distal tibia (38 cases), Phalanges of fingers (4 cases), Distal femur (3 cases), Proximal tibia (3 cases), Phalanges of toes (2 cases), Metatarsal bones (2 cases), Distal radius (2 cases), Distal ulna (1 case), Distal fibula (1 case). Salter-Harris type III (25 cases), including Distal tibia (11 cases), Distal fibula (8 cases), Phalanges of fingers (2 cases), Proximal tibia (2 cases), Distal ulna (1 case), Distal radius (1 case). Salter-Harris type I (14 cases), including Distal radius (3 cases), Phalanges of fingers (3 cases), Distal humerus (2 cases), Distal tibia (2 cases), Phalanges of toes (2 cases), Distal ulna (1 case), Distal fibula (1 case). Salter-Harris type V (1 case), including Phalanges of toes (1 case). (Figs. 1, 2 and 3).

**Table 3:** The epidemiology of age group according to different fractures sites

Fractures sites	Infants	Preschool children	School children	Adolescents
Proximal humerus	1	3	7	11
Distal humerus	51	77	14	4
Proximal radius	0	2	1	0
Distal radius	0	20	193	247
Distal ulna	0	0	11	17
Metacarpal bones	1	2	4	8
Phalanges of fingers	12	81	82	58
Distal femur	7	8	2	0
Proximal tibia	1	1	2	11
Distal tibia	2	16	46	75
Distal fibula	1	5	6	7
Metatarsal bones	1	9	3	1
Phalanges of toes	5	18	12	1

The most common site of epiphyseal fractures was the distal radius (460 cases), in which Salter-Harris type II fractures were the most common (454 cases), followed by Salter-Harris type I (3 cases), Salter-Harris type IV (2 cases), Salter-Harris type III was the least common (1 case). Fractures of the distal radius epiphysis occurred most frequently in the adolescent group (247 cases). The second-most common sites of epiphyseal fractures were the phalanges of the fingers (233 cases), in which Salter-Harris type II fractures were the most common (224 cases) and Salter-Harris type IV (4 cases), Salter-Harris type I (3 cases), Salter-Harris type III fractures were the least common (2 cases). Epiphyseal fractures of the phalanges of the fingers occurred most frequently in the schoolchildren group (82 cases). Next, our findings showed 146 cases of epiphyseal fractures of the distal humerus, in which Salter-Harris type II fractures were the most common (95 cases), followed by Salter-Harris type IV (49 cases), Salter-Harris type I fractures were the least common (2 cases). Epiphyseal fractures of the distal humerus most frequently occurred in the preschool children group (77 cases). In addition, our research showed 139 cases of distal tibia epiphyseal fractures, in which Salter-Harris type II fractures were the most common (88 cases), followed by Salter-Harris type IV (38 cases), Salter-Harris type III (11 cases), Salter-Harris type I fractures were the least common (2 cases). Fractures of the distal tibial epiphysis occurred most frequently in children in the adolescent group (75 cases; Figs. 2 and 3).

### Causes of injuries and hydrological data

The most common cause of injuries leading to epiphyseal fractures in children in this study was falls (720 patients), followed by car accident injuries (68 patients), basketball injuries (43 patients), clipping injuries (38 patients), and crush injuries (36 patients). The causes of injuries also showed different characteristics across different age groups.

Falls were the most common cause of in all age groups, including 274 children in the schoolchildren group, 265 children in the adolescent group, 134 children in the preschool children group, and 47 children in the infant group.

In the infant groups, the second-most common cause of injury was car accidents (9 patients) and clipping (9 patients). And the subsequent injury causes were crush injuries (5 patients), furniture-related falls (4 patients), unknown (2 patients), sprains (1 patient), bunk bed falls (1 patient), falls from height (1 patient), cuts (1 patient), bicycle-spoke injuries (1 patient).

In the preschool children groups, the second-most common cause of injury was car accidents (23 patients). The subsequent injury causes were clipping (22 patients), crush injures (17 patients), bicycle-spoke injuries (12 patients), furniture-related falls (6 patients), cuts (4 patients), unknown (4 patients), twist injuries (3 patients), falls from height (3 patients), slide falls (2 patients), ice skating falls (2 patients), sprains (1 patient), bunk bed falls (1 patient), bicycle falls (1 patient), basketball falls (1 patient), trampoline falls (1 patient), playground falls (1 patient).

In the schoolchildren group, car accidents were the second-most common cause of injuries (20 patients), followed by bicycle falls (9 patients), crush injuries (9 patients), single or parallel bar falls (9 patients), clipping (5 patients), furniture-related falls (5 patients), basketball falls (5 patients), falls while running (5 patients), sprains (4 patients), skateboard falls (4 patients), soccer falls (4 patients), bicycle-spoke injuries (3 patients), kick injuries (3 patients), falls during physical education activities (3 patients), bunk bed falls (2 patients), trampoline falls (2 patients), balance bike falls (2 patients), dance falls (2 patients), falls from height (1 patient), cuts (1 patient), falls from vehicles (1 patient), slide falls (1 patient), ice skating falls (1 patient), playground falls (1 patient), Abuse injuries (1 patient).

In the adolescent group, the second-most common cause of injuries was basketball falls (37 patients). Next were bicycle falls (21 patients), single or parallel bar falls (20 patients), falls while running (17 patients), car accident injuries (16 patients), sprains (7 patients), skateboard falls (6 patients), soccer falls (5 patients), falls during physical education activities (5 patients), crush injuries (5 patients), falls from height (4 patients), kick injuries (3 patients), balance bike falls (3 patients), clipping (2 patients), furniture-related falls (2 patients), falls from vehicles (2 patients), ice skating falls (2 patients), strains (1 patient), dance falls (1 patient), slide falls (1 patient), taekwondo falls (1 patient), jump rope falls (1 patient), unknown (1 patient) (Table 4).

**Table 4:** The epidemiology of age group according to different etiologies

Causes of fractures	Infants	Preschool children	School children	Adolescents
***Daily-life injuries***				
falls	47	134	274	265
crush injuries	5	17	9	5
clipping	9	22	5	2
furniture-related falls	4	6	5	2
sprains	1	1	4	7
bunk bed falls	1	1	2	0
falls from height	1	3	1	4
cuts	1	4	1	0
strains	0	0	0	1
twist injuries	0	3	0	0
***Road traffic injuries***				
car accident injuries	9	23	20	16
bicycle falls	0	1	9	21
bicycle-spoke injuries	1	12	3	0
falls from vehicles	0	0	1	2
***Sports injuries***				
single/parallel bar falls	0	0	9	20
basketball falls	0	1	5	37
falls while running	0	0	5	17
skateboard falls	0	0	4	6
soccer falls	0	0	4	5
kick injuries	0	0	3	3
falls during physical education activities	0	0	3	5
trampoline falls	0	1	2	0
balance bike falls	0	0	2	3
dance falls	0	0	2	1
slide falls	0	2	1	1
ice skating falls	0	2	1	2
playground falls	0	1	1	0
taekwondo falls	0	0	0	1
jump rope falls	0	0	0	1
***Abuse injuries***	0	0	1	0
***Unknown***	2	4	0	1

At the time of injuries in the 1124 observed cases, the average air temperature was 23.7 °C ± 4.4 °C; the average precipitation was 152.56 ± 148.72 mm and the average sunshine duration was 160.45 ± 48.00 h.

### Treatment methods

Of the 1124 children with epiphyseal fractures, 66 did not receive surgical treatment. Among the 66 children who did not receive any surgical, 64 received external plaster fixation alone and two underwent fracture site immobilization. Among the children who had a surgical treatment, 169 underwent open reduction, while 889 underwent closed reduction; the ratio of open to closed reduction was 1:5.3. Moreover, among the 1058 children who received surgical treatments, 884 were treated under general anesthesia, and the remaining 174 received local anesthesia.

Among the 169 cases treated with open reduction, 130 involved Kirschner wire fixation; 30, cannulated screw fixation (screw fixation); 9, unknown. Among the 889 cases treated with closed reduction, 773 involved Kirschner wire fixation; 63, cannulated screw fixation (screw fixation); 53, unknown. (Table 5).

**Table 5:** Treatment and anesthesia

Parameter	Patients
***Non-surgical treatment***	
Fracture site immobilization	2
External plaster fixation	64
***Open reduction subgroup***	169
Kirschner wire fixation	130
Screw fixation	30
Unknown	9
***Closed reduction subgroup***	889
Kirschner wire fixation	773
Screw fixation	63
Unknown	53
***Local anesthesia***	174
***General anesthesia***	884

### Interval between the injury and hospital visit

In our study population, the interval between injury and hospitalization was categorized as < 4 h (389 patients), 4–6 h (405 patients), 7–11 h (92 patients), 12–23 h (56 patients), 24–47 h (61 patients), 48–72 h (33 patients), 73 h to 5 d (27 patients), and 6–14 d (61 patients).

The interval between injury and the hospital visit was > 72 h in 88 patients, including 53 boys and 35 girls. The most common cases in the adolescent group were 36 patients, followed by 25 patients in the schoolchildren group, 20 patients in the preschool children groups, and 7 patients in the infant groups. All these 88 children were treated with surgery, including 57 cases of closed reduction and 31 cases of open reduction. (Table 6).

**Table 6:** The interval between injury and hospitalization

Interval Time	Patients
< 4 h	389
4–6 h	405
7–11 h	92
12–23 h	56
24–47 h	61
48–72 h	33
73 h to 5 d	27
6–14 d	61
The interval between injury and the hospital visit was > 72 h	88
Boys	53
Girls	35
Infants	7
Preschool children	20
School children	25
Adolescents	36
Closed reduction	57
Open reduction	31

Among these 88 patients, the most common fracture type was a distal radial epiphyseal fracture (32 cases), all of which were of Salter-Harris type II. The main reason for delayed hospitalization was delayed transfer to our hospital after unsatisfactory treatment results in other hospitals not specialized for children (74 cases). Among the remaining 14 cases, our outpatient conservative treatment failed and hospital admission was required in 7 cases; the fractures were ignored by family members in 5 cases; and family members refused hospitalization in 2 cases.

## Discussion

This study yielded some important findings: (1) Regarding epiphyseal fractures, boys were more often hospitalized than girls in all age groups, and the adolescent group included the largest number of hospitalized children as well as the highest male-to-female. (2) Among all 1147 epiphyseal fractures, Salter-Harris II fractures were the most common, followed by Salter-Harris IV fractures. (3) The most common epiphyseal fracture site was the distal radius, in which Salter-Harris type II fractures were the most common, and most frequently occurred in the adolescent group. (4) The most common cause of epiphyseal fractures was falls, followed by car accident injuries. (5) Surgery was the primary treatment modality for epiphyseal fractures, and the ratio of open to closed reduction was 1:5.3. (6) Delayed diagnosis and an interval > 72 h between injury and hospitalization was observed in 88 patients. Among the cases involving delayed treatment, the most common type of fracture was distal radial epiphyseal fracture, all of which were of Salter-Harris type II.

### Age and sex

Fractures of the epiphysis occur at all ages until epiphyseal closure. The incidence of epiphyseal fractures increases gradually in boys and girls from birth to 11 years of age, continues to increase in boys aged 14 years, but declines rapidly in girls from 13 years and in boys from 15 years of age [9–14]. The study by Kawamoto et al. showed that the overall incidence of epiphyseal injuries peaked at the age of 12 years in both boys and girls, with an average age of 10.3 years for boys and 8.9 years for girls [3]. Peterson et al. reported that epiphyseal injuries peaked at 14 years of age in boys and 11 or 12 years of age in girls [15]. In our study, epiphyseal fractures were most commonly observed in girls aged 7–11 years and boys aged 12–18 years.

Morscher et al. summarized that 68%–85% and 15%–32% of epiphyseal fractures occurred in boys and girls, respectively (ratio, 5.6–2.1:1) [16]. In our results, the sex ratio among all children with epiphyseal fractures was 2.4:1, and the highest sex ratio (5.3:1) was observed in the adolescent group. Although adolescent boys have more prominent and stronger bones, they are more likely to experience fractures than girls because of their preference for more intense, competitive, and confrontational activities. Therefore, health education activities should focus on safety education for boys, especially adolescent male children and those who are active and like to participate in high-risk sports that may cause serious injuries.

### Fracture site and classification

Upper-extremity epiphyseal fractures have been reported to be more frequent than lower-extremity fractures [13]. Our findings also showed 907 epiphyseal fractures in the upper extremities in comparison with 240 fractures in the lower extremities; the higher number of epiphyseal fractures in the upper extremities may be connected to fractures caused by fall-related traumatic energy. As for the epiphyseal fracture site, the most common area has been reported to be the phalanx, followed by the distal radius [13, 15]. Similarly, Japanese studies have reported that the most common epiphyseal fracture site is the phalanx, with most cases showing Salter-Harris type II fractures (73.8%) [3]. In contrast, our findings indicated that the most common site of epiphyseal fractures was the distal radius followed by phalanges of the fingers, which was consistent with the findings of other previous studies [10, 12, 17, 18].

A single-center study of patients from 1972 to 1983 found that the epiphysis was involved in 28% of children with distal radius fractures. They also found a lower rate of Salter-Harris type II fractures than Salter-Harris type I [10]. Similarly, two studies reviewing radial fractures in children found that only 10% [19] and 18% [20] involved the distal radial epiphysis. However, in a study of wrist fractures in children, 54% involved the distal radial epiphysis [21]. Most of the distal radial epiphyseal fractures were of Salter-Harris type II (84.3%), and the rest were of Salter-Harris type I (11.1%), type III (0.4%), and type II (3.4%) [22]. A Japanese study also found that most of the distal radius epiphyseal fractures were of Salter-Harris type II (89.2%) [3]. Our findings also showed that Salter-Harris type II was the most common (98.7%) categorization for distal radius epiphyseal fractures. Distal radius epiphyseal fractures can result in growth disorders and mechanical changes in the wrist joint, which can greatly impact the growth and development of children. Therefore, physicians should examine the wrist in detail. The standard clinical features of distal radial epiphyseal fractures are a history of trauma, wrist swelling and tenderness, and reluctance to move the wrist and fingers [23–25].

Among lower-extremity epiphyseal fractures, distal tibial epiphyseal fractures are more common than fractures at other lower-extremity sites [26], and Salter-Harris type II fractures are the most common, accounting for 40% of the distal tibial fractures in children [15]. In addition, the incidence of premature physeal closure in Salter-Harris type II distal tibial epiphyseal fractures can be as high as 67% [27]. In our study, the distal tibia was the most common site of lower-extremity epiphyseal fractures, and most of these fractures were categorized as Salter-Harris type II (63.3%). The mechanism of distal tibial epiphyseal fractures usually involves an abnormal torsional force and fixation of the foot in a position opposite to that produced by the mechanism. These fractures show the highest propensity for complications [28, 29] and the most common one is the premature epiphyseal block, which impairs average longitudinal growth, resulting in angular deformity and limb length inequality.

### Causes of injuries

Falls are the most likely cause of epiphyseal fractures, usually associated with running or falling from furniture, playground equipment, or trees; falls are followed by competitive sports (football, basketball, gymnastics, hockey, baseball, wrestling, softball, volleyball, and track and field items), which accounted for 33.5% of all cases, and motor vehicle accidents involving cars, trucks, buses, motorcycles, and all-terrain vehicles, which accounted for 5% of fractures [15]. Other studies also identified falls as the leading cause of fractures in children [8, 30, 31]. In our study, among the infant, preschool, and schoolchildren groups, the two most common causes of major injuries were falls and car accident injuries. In contrast, among children in the adolescent group, the two most common causes of injury were falls and basketball falls. Children's lively and active nature and the improvement in their motor ability with age can often result in falls during play and sports, and children’s fractures caused by falls usually occur at home (53%), schools (16.5%), and amusement parks (10%) [32]. Thus, children engaged in playing and sports activities in these sites should receive close attention to prevent falls or other accidental injuries and reduce the occurrence of epiphyseal fractures.

Regarding motor vehicle accidents, a study from Malaysia reported that motor vehicle accidents were the second leading cause of injury (30.7%) among children hospitalized for trauma [33]. Canadian researchers collected data on 237 children with severe trauma between 1996 and 2000 and found that the most common cause was a traffic accident (39%) [34]. The high-energy trauma of a motor vehicle accident, which is often associated with open fractures, polytrauma, and shock, poses a severe health risk to children. Therefore, parents, communities and schools should receive education on traffic safety and strengthen the supervision of children's outdoor activities.

### Strengthening the understanding of epiphyseal fractures in children

The musculoskeletal system in children is in a state of continuous development and maturation, and children’s bones are not merely smaller versions of adult bones. Epiphyseal injury is a unique type of injury in pediatric fractures [35], and the diagnosis and treatment of such fractures by non-pediatric orthopedic specialists is associated with significant pitfalls, including missed diagnosis, misdiagnosis or improper treatment, which can have catastrophic consequences. For example, incorrect diagnoses and treatment measures can affect the growth and development of the child’s bones and, in severe cases, cause premature epiphyseal closure and even the formation of bone bridges [36], resulting in varus or valgus deformities of the limbs. Moreover, epiphyseal fractures that do not receive standardized treatment may cause cessation of limb development, resulting in obvious limb deformities or length differences [37], limited joint mobility and even lifelong disability, imposing substantial limitations in the patient’s regular work and life.

In our study, the main reason for intervals > 72 h between the injury and hospitalization was that the patients were first seen at other hospitals not specialized for children and they were subsequently transferred to our hospital because of unsatisfactory treatment effects at the previous hospital. On the basis of these findings, we recommend strengthening the basic knowledge of pediatric orthopedic epiphyseal injuries among emergency department surgeons and orthopedic surgeons in general hospitals [8, 38], especially for the diagnosis and treatment of Salter-Harris II epiphyseal injuries in the distal radius. In addition, for children with clinical manifestations of suspected epiphyseal injury (swelling, pain and mobility impairment), radiographs of the uninjured wrist joint can be taken for comparison or MRI can be performed when necessary to clarify the site and type of epiphyseal injury [39–41].

## Conclusions

The findings highlight the need to strengthen safety education and improve protective measures for children's activities depending on the age, sex, cause of injury, location, type of epiphyseal fracture and other characteristics. In addition, basic knowledge of pediatric orthopedic epiphyseal injuries should be improved among general hospital emergency surgeons or orthopedic surgeons to reduce the possibility of irreversible damage as a result of missed diagnosis, misdiagnosis or improper treatment of epiphyseal fractures. These improvements can be expected to contribute positively to the protection of children's health.

## Data Availability

The data sets used and analysed during the current study are available from these corresponding authors on reasonable request.
